# Risk factors of hepatitis B infection: Health policy makers should be aware of their importance in each community

**Published:** 2011-04-01

**Authors:** Seyed Mohammad Miri, Seyed Moayed Alavian

**Affiliations:** 1 Baqiyatallah Research Center for Gastroenterology and Liver Diseases, Tehran, IR Iran

**Keywords:** Hepatitis B infection, Risk factors, Iran, Turkey, Vaccination, Prevention

Despite the availability of an effective vaccine, Hepatitis B virus (HBV) infection still remains a foremost health problem. Undoubtedly, finding the key routes of hepatitis transmission from the point of prevention in every country, specifically in endemic regions, is of high priority. Such efforts are especially important given that many infected patients with hepatitis are asymptomatic [1][2]. Iran and Turkey are located in the Middle East, and HBV prevalence varies from intermediate to high in most countries in this region. In most countries across the world, the epidemiology of HBV infection has changed after the integration of HBV vaccination in infants and high-risk groups [3][4][5][6]. The most frequent risk factors of HBV infection are familial contact, blood transfusion, hospitalization, surgery, and sexual contact [7][8]. Ozor et al. presented a series of valuable data concerning the risk factors of HBV infection in a general population of Turkey [9]. They described that individuals with close familial contact with HBsAg-positive patients face the greatest risk of acute hepatitis B infection in Turkey. Because Ozor et al. did not find a relationship between the disease and other risk factors, any generalization of the risk factors of acquiring HBV infection to the general population is inappropriate. Most HBV-infected patients are asymptomatic, and acquiring the infection at an early age is generally associated with no symptoms.

HBV is transmitted through both vertical and horizontal routes. Although vertical routes have both been very common in Turkey and Iran, nowadays the horizontal routes are much more important [8]. In other words, Iran and Turkey have many similarities in HBV transmission. Specifically, although familial contact with HBV-infected patients may play an important role in horizontal HBV transmission, the vertical transmission still remains as a prominent role in infected families [7][8]. The transition from vertical to horizontal transmission of HBV has indeed occurred, and, consequently, we should change our strategy for controlling HBV infection in our communities. Sexual transmission is the most important mode of HBV transmission in several developed countries, and it is an important risk factor of HCV and HBV infection in Iran and Turkey [7][8][10][11]. Some groups, such as health-care workers, especially surgeons, nurses, and dentists; policemen; barbers; and drivers, are at higher risk of acquiring HBV infection in our region [7]. Barbers are a high-risk group for HBV infection in Turkey as well [12]. The main goal in determining high-risk occupations is preventing HBV transmission among these groups.

Other studies recommend the extension of HBV vaccinations in infant and high risk group, screening during pregnancy, and implementing additional strategies Such as adding the injection of hepatitis B immunoglobulin (HBIG) to the routine vaccination in neonates of mother HBs Ag positive, extension of vaccination in adults [1][13][14][15]. Consequently, after several years it seems that the primary vertical route-mothers-has shifted to horizontal routes of HBV transmission, particularly in our region.
